# Isolated Meniscal Ramp Lesion Without Obvious Anterior Cruicate Ligament Rupture

**DOI:** 10.1111/os.12860

**Published:** 2020-12-14

**Authors:** Jun Jiang, Lei Ni, Jian Chen

**Affiliations:** ^1^ Arthritis Clinic and Research Center Peking University People Hospital Beijing China

**Keywords:** All‐inside horizontal mattress suturing, Isolated ramp lesion, Knee arthroscopy, Without obvious ACL rupture

## Abstract

**Objectives:**

The purpose of the present paper was to study isolated meniscal ramp lesions without obvious ACL rupture. Their biomechanical mechanisms were analyzed and their clinical characteristics were reviewed. The clinical effects of an all‐inside horizontal mattress suture for isolated ramp lesions were evaluated.

**Materials and Methods:**

Twenty isolated meniscal ramp lesion patients without obvious ACL rupture from 2015 to 2017 were retrospectively reviewed. Preoperative MRI showed intact ACL and signs of ramp lesions. These isolated ramp lesions were arthroscopically confirmed and repaired through an all‐inside horizontal mattress suturing method. MRI was performed 3 months postoperatively to assess isolated ramp lesion healing. The Tegner–Lysholm score and the visual analog scale score were recorded preoperatively and at 2 years postoperatively. The Wilcoxon rank sum test was performed to determine statistical significance.

**Results:**

Arthroscopic exploration confirmed isolated ramp lesions and longitudinal ACL splits or degeneration without obvious ACL rupture. MRI 3 months postoperatively showed healing of the isolated ramp lesions. At 2 years postoperatively, the VAS scores were significantly decreased and the Tegner–Lysholm scores were significantly increased. Knee function, without pain, was restored in all patients, including walking, climing and descending stairs, and squatting.

**Conclusion:**

Isolated meniscal ramp lesions without obvious ACL rupture may exist because of ACL longitudinal splits or degeneration and can be repaired through anterolateral and anteromedial portals with an all‐inside horizontal‐mattress suturing method.

## Graphical abstract

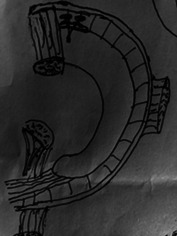

All‐inside horizontal mattress suture method with the 12° OMNISPAN Meniscal Repairing System for isolated ramp lesions without obvious ACL rupture in the right knee.

## Introduction

A ramp lesion (or “hidden” lesion), a tear of the peripheral meniscocapsular attachments of the posterior horn of medial meniscus (PHMM), is usually concurrent with acute anterior cruicate ligament (ACL) rupture or occurs following chronic ACL laxity after ACL rupture. The term “ramp lesion” was first described by Strobel in the 1980s^1^ and is useful for differentiating these lesions other types of longitudinal posterior horn tears. These lesions have been reported in 15%–17% of patients undergoing an ACL reconstruction^2^, ^3^, ^4^.

To our knowledge, there are no reports in the published literature of isolated ramp lesions without obvious ACL rupture. However, isolated ramp lesions without obvious ACL rupture have been observed in our clinical practice. The present paper analyzes the biomechanical mechanisms of isolated ramp lesions without obvious ACL rupture. Clinical characteristics and the arthroscopic repair method and its clinical effects for these patients were retrospectively reviewed.

## Materials and Methods

From 2015 to 2017, 20 patients with isolated meniscal ramp lesion without obvious ACL rupture were treated in our clinic. The clinical data of these patients was retrospectively reviewed.

Inclusion criteria: (i) patients with posteromedial knee pain and flexion limitation, signs of medial joint line tenderness posteriorly, and positive McMurray tests during extreme flexion and external rotation, without obvious anterolateral laxity; (ii) indication on MRI of a ramp lesion (i.e. high signal irregularity of the capsular margin or separation in the meniscocapsular junction of the PHMM on T2‐weighted sagittal images); and (iii) ACL intact in MRI.

Exclusion criteria: (i) severe knee osteoarthritis with obvious varus and/or flexion contracture deformity; (ii) knee rheumatoid arthritis; (iii) knee septic arthritis; and (iv) patient with posteromedial knee pain and flexion limitation with obvious complete or partial ACL rupture on MRI.

A total of 3 male patients and 17 female patients were included in the study, with a mean age of 56.9 years (27–75 years old).

**Fig. 1:**
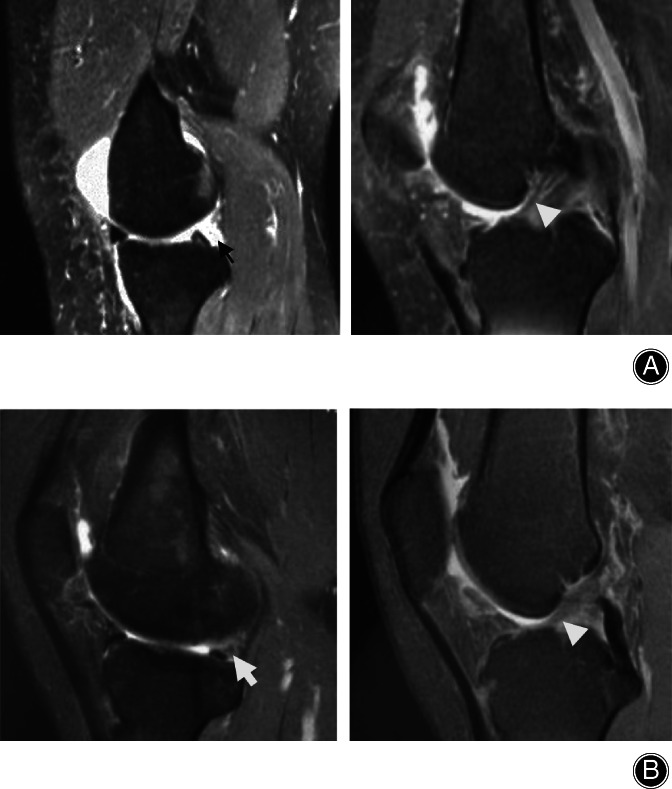
A 36‐year‐old female patient suffered from posteromedial left knee pain without obvious anterior cruicate ligament (ACL) rupture. (A) Preoperative fat suppression (FS) T2 MRI sagittal image shows isolated ramp lesion of the posterior horn of medial meniscus (PHMM) (black arrow) with intact but degenerative ACL integrity (white triangle), manifesting as high signal fluid between the PHMM and the posterior capsule. (B) After 6 months, postoperative FS T2 MRI sagittal images show healing of the ramp lesion (white arrow), manifesting as low signal meniscocapsular attachment tissue of the PHMM.

**Fig. 2:**
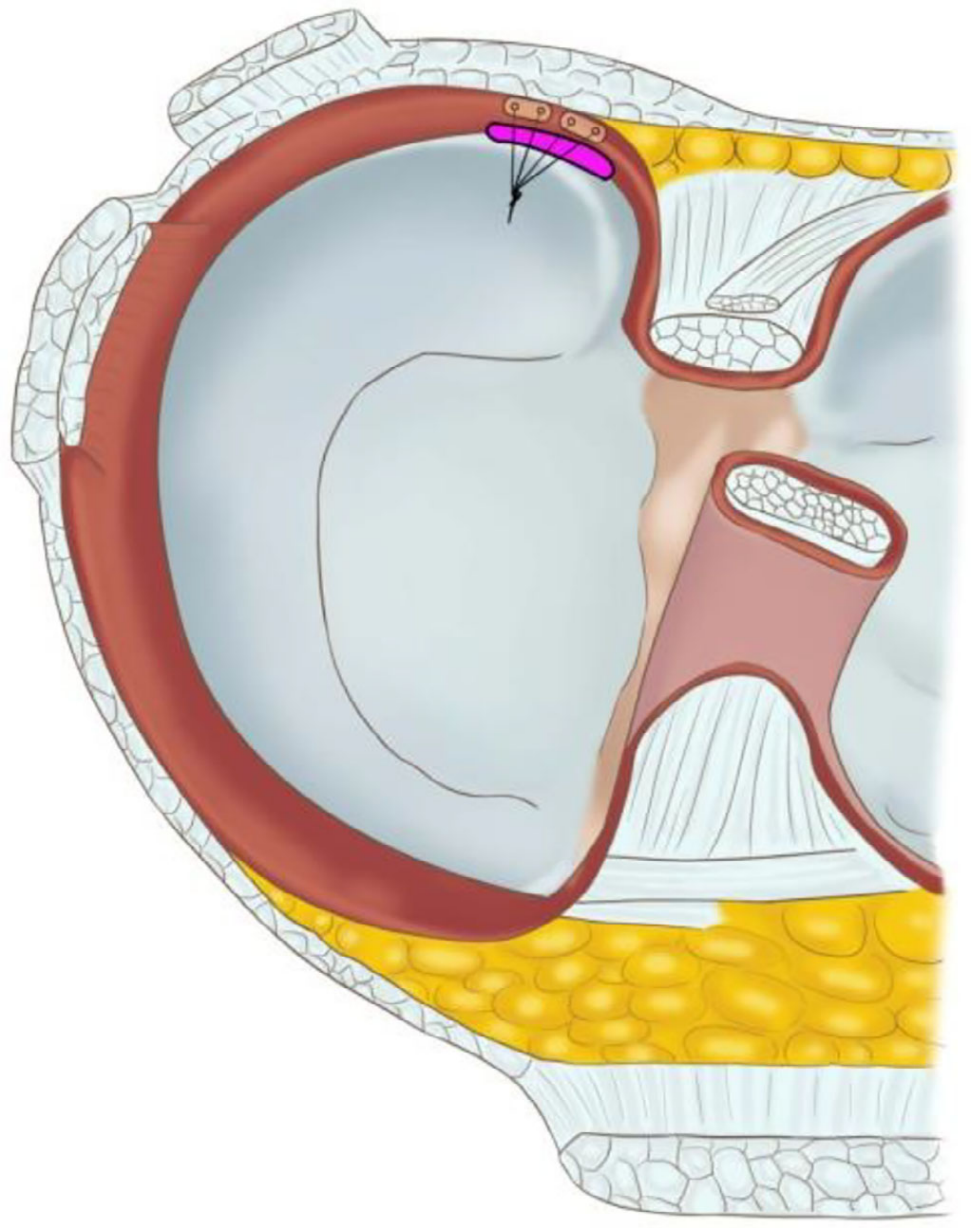
Schematic drawing of horizontal mattress suture method with 12° OMNISPAN Meniscal Repair System for an isolated ramp lesion without obvious anterior cruicate ligament rupture in the left knee.

MRI was performed preoperatively (Fig. 1). A ramp lesion was arthroscopically confirmed with posteromedial portal probing through intercondylar notch viewing or posteromedial portal viewing and repaired using an all‐inside horizontal mattress suturing method (Fig. 2) with the 12° OMNISPAN Meniscal Repair System (Depuy Mitek, Rayhanm MA, USA) through anterolateral and anteromedial portals. Patients were evaluated postoperatively at 6 weeks and at 3, 6, 12, and 24 months in an outpatient clinic. The mean follow‐up period was approximately 26 months. MRI was performed (Fig. 1). The visual analogue scale (VAS) score and the Tegner–Lysholm score were recorded preoperatively and at the last follow up. The Wilcoxon rank sum test was performed to determine the statistical significance between the preoperative results and those at the final follow up.

Visual analog scale score: The VAS is a unidimensional measure of pain intensity in the adult population. The score is determined by measuring the distance (cm) on a 10‐cm line (with 1 mm in one unit) between the “no pain” anchor and the patient's mark, providing a range of scores from 0 to 10. For pain intensity, the scale ranges from “no pain” (score of 0) to “pain as bad as it could be” or “worst imaginable pain” (score of 10). The cutoff points for VAS are: no pain (0–0.4 cm), mild pain (0.5–4.4 cm), moderate pain (4.5–7.4 cm), and severe pain (7.5–10 cm).

The Tegner–Lysholm score: The Tegner–Lysholm score is used to evaluate activities of daily living scales for patients with a variety of knee disorders, including ligament and meniscus injuries and patellofemoral pain. Eight factors are rated to produce an overall score on a point scale of 0 to 100. The factors of limp, support, and squatting are worth a potential of 5 points each, pain and instability 25 points each, swelling and stair climbing 10 points each, and locking 15 points. An assignment is given as “excellent” for 95 to 100 points, “good” for 84 to 94 points, “fair” for 65 to 83 points, or “poor” for less than 65 points.

### *Surgical Method*

**Fig. 3:**
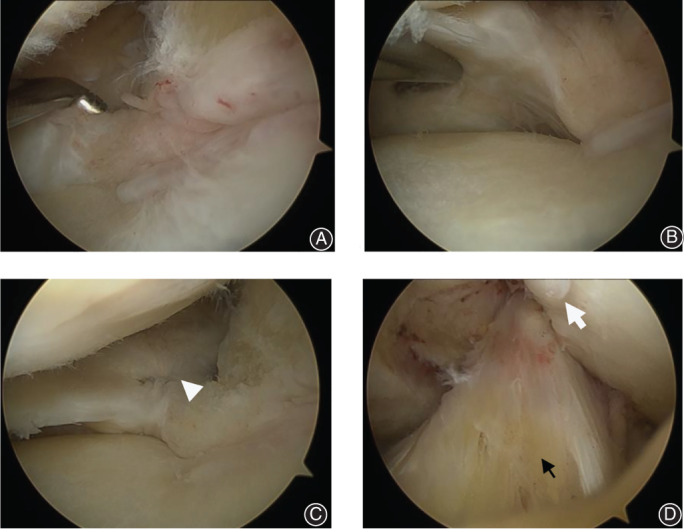
(A) Ramp lesion of the posterior horn of medial meniscus in the left knee of a 71‐year‐old female patient was viewed through the anterolateral portal. The hook tip can be inserted into the meniscocapsular junction, which shows a meniscocapsular, tear (i.e. a ramp lesion). (B) Probe examination from inferior surface shows the menisco‐tibial ligament intact. (C) The view from the anterolateral portal shows an all‐inside horizontal mattress repair with a 12° OMNISPAN Meniscal Repair System from the upper surface of a ramp lesion (white triangle). (D) The view from the anterolateral portal shows anterior cruicate ligament longitudinal split (black arrow) and intercondylar fossa osteophyte (white arrow).

**Fig. 4:**
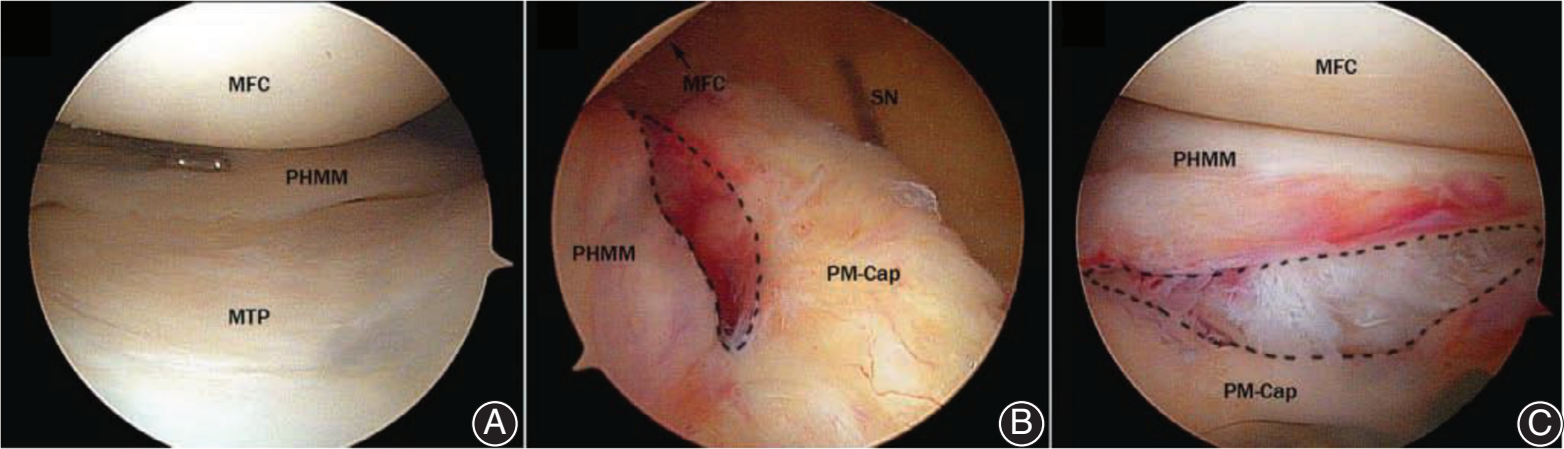
Arthroscopic view of a ramp lesion in a left knee. A, View through the anterolateral portal; the PHMM seems normal. B, View through intercondylar notch; the dashed area shows the ramp lesion. C, View through the posteromedial portal. MFC, medial femoral condyle; MTP, medial tibial plateau; PM‐Cap, posteromedial capsule; SN, spinal needle; PHMM, posterior horn of the medial meniscus.

The patient was placed in the supine position for the operation and given lumbar anesthesia. The knee joint was examined through the anterolateral portal with a 30^°^ arthroscope by an experienced arthroscopic surgeon. The ACL integrity and tension was routinely checked through arthroscopic examination and probe tensioning. Some longitudinal splits were observed without obvious complete or partial ACL rupture. In order to enlarge the posteromedial vision, the medial collateral ligament was pie‐crust released with a 5‐mL syringe needle in the joint line. Otherwise, the view of the PHMM may have been partly blocked by the medial femoral condyle. With the knee placed in valgus and hyperextension position, examination of the PHMM was performed, with meticulous probing, to find the ramp lesion. Once the probe hook tip (4 mm in length) can be inserted into the meniscocapsular junction (meniscofemoral ligament) from the upper surface of the medial meniscus, the ramp lesion of the PHMM can be observed (Fig. 3). Because of the tension of the medial collateral ligament and the convex contour of the medial condyle, it is not easy to explore ramp lesions arthroscopically through the anterolateral portal. Usually, we explore and confirm ramp lesions through the intercondylar notch view (between the medial condyle and the posterior cruciate ligament). To visualize the posteromedial compartment for inspection of the meniscocapsular junction area, a 30° arthroscope was advanced through the intercondylar notch space^5^. A 70° arthroscope may be more helpful through enabling wider vision. The posteromedial portal was created with a 5‐mL syringe needle insertion into the posteromedial compartment. The meniscocapsular junction of the PHMM was examined with a probe to detect ramp lesions through the posteromedial portal. The meniscocapsular junction of the PHMM may be easily visualized and confirmed through posteromedial view (Fig. 4). We used a 12° OMNISPAN Meniscal Repair System to perform an all‐inside horizontal mattress repair through the anteromedial portal with an anterolateral view. To facilitate the repair procedure, the knee was placed in valgus and hyperextension position to avoid blocking of the medial femoral condyle to the needle in the OMNISPAN applier. Medial collateral ligament pie‐crusting is also helpful in performing the all‐inside repairing procedure. Although potentially difficult, we successfully performed all these ramp lesion repairs using the method described.

### *Rehabilitation Procedure*

Usually, patients use a knee brace for approximately 3 months. At 6 weeks postoperatively, the knee is limited to a 0° extension position. After 6 weeks, the knee is limited to a 60° flexion range. On the first day postoperatively, the patient can begin straight leg raising exercises to improve quadriceps muscle strength and knee flexion/extension exercises on a bed. The knee flexion range is increased gradually on a bed. After 2 weeks, knee flexion can be increased to more than 90°. After 3–4 weeks, knee flexion can be increased to more than 120°. On the first day postoperatively, the patient can walk with partial weight‐bearing with a knee brace and crutches. At 3 months postoperatively, with MRI demonstrating healing of the RAMP lesion, the patient can walk with full weight‐bearing without a knee brace and crutches. Squatting and jogging were permitted 4 months postoperatively. Following normal single leg hop for a distance (with the distance hopped on the involved leg more than 85% of the uninvolved leg) and negative Thessaly test, pivot activity was permitted at 6 months and full activity at 9 months.

## Results

After 3 months, these patients have no posteromedial pain in the knee. They can walk without a knee brace and crutches. After knee rehabilitation exercises, full range of motion can be achieved. However, pivot activity should be avoided until 6 months postoperatively to prevent retearing of the ramp area. Following normal single leg hop for a distance and a negative Thessaly test, pivot activity was permitted at 6 months and full activity at 9 months.

### *Visual analogue scale and* *Tegner–Lysholm* *score*

The VAS score was decreased from 6.5 (±1.5) points preoperatively to 2.1 (±1.4) points 2 years postoperatively. The Tegner–Lysholm knee function score was increased from 35.6 (±5.3) points preoperatively to 85.7 (±3.5) points 2 years postoperatively. Both have statistical significance (*P* < 0.01) using the Wilcoxon rank sum test (SPSS 20.0, SPSS, Chicago, IL, USA), as shown in Table 1.

**TABLE 1:** Visual analogue scale (VAS) and Tegner–Lysholm score (*n* = 20, *X* ± *S*)

	VAS score	Tegner–Lysholm score
Preoperative	6.5 ± 1.5	35.6 ± 5.3
Postoperative	2.1 ± 1.4	85.7 ± 3.5
*t*‐value	9.96	73.58
*P*‐value	0.002	0.003

Preoperatively, high signal irregularity of the capsular margin or separation in the meniscocapsular junction of the PHMM is shown on T2‐weighted sagittal images. Postoperative MRI examination at 3 or more months shows healing of a ramp lesion, manifesting as low signal meniscocapsular attachment tissue of the PHMM. (Fig. 1).

## Discussion

Meniscal ramp lesions, defined as a peripheral detachment lesion of the PHMM, usually occur at the time of acute ACL rupture and in knees with chronic ACL laxity following ACL rupture, which significantly increases with time until 24 months after the initial ACL injury. Patients younger than 30 years of age and male patients are more susceptible to meniscal ramp lesions^3^.

Anterior cruicate ligament ruptures increase tibial anterior translation and internal rotation, which cause the medial meniscus to “engage” the posteromedial femoral condyle and act as a wedge against the postero‐medial tibia^6^, ^7^, ^8^. However, Hughston^9^ suggested that semimembranosus muscle contraction with the wedge effect of the PHMM would produce great stress at the meniscocapsular junction and possibly result in a peripheral meniscocapsular longitudinal tear (ramp lesion).

In our clinic, isolated meniscal ramp lesions without obvious ACL rupture were observed in some patients. ACL longitudinal splits were observed arthroscopically in these patients. An important suspicious finding during probe examination is probe insertion into the menicsocapsular junction despite the lack of a visible lesion in PHMM.

Anterior cruicate ligament longitudinal splits were observed intraoperatively with probe tensioning in these patients. In young patients, ACL longitudinal splits may be caused by daily knee pivot activity or hyperextension. In older patients, ACL longitudinal splits may be caused by degenerative osteoarthritis, which can lead to impingement and attrition of the ACL due to intercondylar fossa osteophytes. We speculate that ACL longitudinal splits and degeneration cause minor anterior instability. To resist the minor instability, semimembranosus muscle will contract. Minor instability and semimembranosus muscle contraction may cause a wedge effect of the PHMM with the posteromedial femoral condyle and increase stress in the meniscocapsular junction of the PHMM, which would gradually result in an isolated ramp lesion without obvious ACL rupture.

The PHMM is known to have a secondary effect of limiting anterior translation of the tibia^10^. Ramp lesion repair significantly increases postoperative knee function following ACL reconstruction^11^. Peltier^12^ concluded that ramp lesions play a significant role in knee stability and also increase the tension forces in the ACL. Numerous other investigators have demonstrated that in the presence of a ramp lesion, isolated ACL reconstruction fails to restore normal joint kinematics and results in residual laxity^5^, ^12^, ^13^. Furthermore, it has been demonstrated that repair of ramp lesions abolishes^13^, ^14^, ^15^ the pathologic increase in laxity, providing a biomechanical rationale for identifying and repairing these lesions.

Isolated ramp lesions without obvious ACL rupture should also be repaired to improve knee function. When a patient has posteromedial knee pain and limited flexion or inability to squat, this will cause a surgeon to be suspicous of a ramp lesion. If, based on physical examination, a patient has a negative Lachman test or anterior drawer test, an isolated ramp lesion without obvious ACL rupture should be considered.

Preoperative MRI is necessary to assess the ACL integrity and isolated ramp lesions. Correct diagnosis of a ramp lesion on MRI would enable more accurate planning of treatment (e.g. consideration of using an additional posteromedial portal)^16^. MRI diagnosis for ramp lesions required high signal irregularity of the capsular margin or separation in the meniscocapsular junction of the PHMM on T2 sagittal images. The finding of posterior marginal irregularity may reflect scarring made by separation of the PHMM and the posterior capsule, and fluid filling may reflect the space created by separation of the PHMM and the posterior capsule^17^. Arner *et al*. reported moderate to high sensitivity and excellent specificity in detecting meniscal ramp lesions on MRI. While MRI is effective in identifying posteromedial meniscocapsular injuries, arthroscopic evaluation remains the gold standard^18^. Based on patient symptoms, physical examination, and MRI, isolated ramp lesions without obvious ACL rupture may be diagnosed preoperatively.

According to Sonnery–Cottet classification^19^, an isolated ramp lesion without obvious ACL rupture is a meniscocapsular rupture without meniscotibial ligament disruption (type 1). These lesions are very peripherally located in the synovial sheat of the posteromedial capsule. Their mobility at probing is very low. These characteristics make it possible to perform an all‐inside isolated ramp lesion repair with the OMNISPAN Meniscal Repair System through routine anterolateral and anteromedial portals. Because of the longitudinal rupture of an isolated ramp lesion, a horizontal mattress suture can be performed to restore good stability after repairing. After 3 months, healing of isolated ramp lesions can be achieved in these patients **(**Fig. 1 **)** because of the good blood supply in the ramp region and good repairing stability.

Arthroscopy is considered the gold standard for diagnosis of ramp lesions^19^. However, it is not without pitfalls. Forty percent of ramp lesions are not identified through standard anterior portal visualization. Inspection of the posterior compartment through intercondylar transnotch view and posteromedial probing are necessary to identify them^19^, ^20^. Improved visualization through intercondylar transnotch view and a posteromedial working portal are critical: (i) to improve diagnosis of ramp lesions^21^; (ii) for better diagnosis through probing and debridement from the posteromedial portal before repair; and (iii) for better control of the complete closure of the ramp lesion^22^.

After diagnosis confirmation through intercondylar transnotch view, debridement from the posteromedial portal before repairing is important. Then horizontal mattress all‐inside suture of meniscocapsular ruptures can be performed through the anteromedial portal and anterolateral view^23^. For longitudinal ACL splits or degeneration, we used radiofrequency to shrink the ACL and increase ACL tension. By applying in conjunction with all‐inside meniscal ramp lesion repair, the minor instability was reduced.

At 3 months postoperatively, MRI was used to confirm healing of meniscocapsular rupture. Improved knee function score and no posteromedial knee pain or flexion/squatting limitation were also confirmed. MRI signal of a healing meniscocapsular rupture manifests as low signal meniscocapsular attachment of the PHMM. Because of the unwillingness of Chinese patients to undergo surgery, we cannot perform second‐look arthroscopic examinations to confirm meniscocapsular rupture healing.

### *Conclusion*

Our study shows that isolated meniscal ramp lesions without obvious ACL rupture may exist because of ACL minor laxity caused by longitudinal splits or degeneration. Performing all‐inside horizontal mattress suturing repair with the OMNISPAN Meniscal Repair System, isolated ramp lesions can be repaired through anterolateral and anteromedial portals. Patients experience improved knee function without posteromedial knee pain.
